# mRNAs sequestered in stress granules recover nearly completely for translation

**DOI:** 10.1080/15476286.2022.2094137

**Published:** 2022-07-07

**Authors:** Sarada Das, Leonardo Santos, Antonio Virgilio Failla, Zoya Ignatova

**Affiliations:** a Institute of Biochemistry and Molecular Biology, University of Hamburg, Hamburg, Germany; b Microscopy Imaging Facility, University Medical Center Hamburg-Eppendorf, Hamburg, Germany

**Keywords:** mRNA, translation, m^6^A modification, deep sequencing, mRNA structure, stress granules

## Abstract

Stress granules (SGs) are membrane-less condensates composed of RNA and protein that assemble in response to stress stimuli and disassemble when stress is lifted. Both assembly and disassembly are tightly controlled processes, yet, it remains elusive whether mRNAs in SGs completely recover for translation following stress relief. Using RNA-seq of translating fractions in human cell line, we found that higher fraction of the m^6^A-modified mRNAs recovered for translation compared to unmodified mRNAs, i.e. 95% vs 84%, respectively. Considering structural mRNA analysis, we found that the m^6^A modification enhances structuring at nucleotides in its close vicinity. Our results suggest that SG-sequestered mRNAs disassemble nearly completely from SGs and the m^6^A modification may display some advantage to the mRNAs in their recovery for translation likely by m^6^A-driven structural stabilization.

## Introduction

Stress granules (SGs) are crucial for cells to sustain and adapt to stress. SGs are dynamic cytoplasmic membrane-less condensates that are composed of RNA and proteins and form via liquid-liquid phase separation in response to various types of stress, including oxidative agents, heat stress, glucose deprivation [1,2]. The assembly of SGs is tightly regulated and misregulation is implicated in several human pathologies [3,4]. SGs assemble through weak and transient protein-protein, RNA-protein and RNA-RNA interactions and when the sum of these interactions reaches a threshold, known as percolation threshold, the extensive interaction network separates the SGs from the surrounding milieu creating a liquid condensate [5–7].

Exposure to stress rapidly inhibits translation initiation and ceases translation, and the following ribosomal run-off raises the influx of unprotected mRNAs capable of mediating RNA-protein or RNA-RNA interactions [8]. SGs are dynamic structures with heterogeneous distribution of their constituents [9], whereby some structural subdivision within SGs is recognized, with a more stable internal core surrounded by a rim, which dynamically exchanges components with translating ribosomes or polysomes [5,10]. Several different proteins have been detected in SGs [10], but only approximately 36 of them together with RNAs provide the core of SG interaction network in establishing the percolation threshold [11]. Posttranslational modifications of the core SG proteins are suggested to be the key to SG assembly (reviewed in [9]). Earlier quantification of the RNA constituents of SGs suggested that only a small portion of the bulk cellular mRNAs assemble into SGs with longer coding sequences (CDS) and UTRs that are mostly inefficiently translating [12], with many mRNAs encoding house-keeping proteins [13]. Other studies propose that the majority of the cellular mRNAs can condensate into SG and the most prevalent mRNA modification, the m^6^A, in some of those mRNAs enhances their phase-separation potential and partitioning into SG though interactions with YTHDF proteins [14,15].

SGs are considered as cytoplasmic condensates of stalled translational preinitiation complexes that accumulate during stress [13]. The mechanism of SG formation and their protein composition vary and are dependent on the stress type or initiation factor involved. Based on this, three different SG subtypes have been identified, e.g. type I – t triggered by stress-related phosphorylation of the eIF2α, type II – formed through inactivation of eIF4A in a eIF2α-phosphorylation-independent manner, and type III – lacking eIF3 (reviewed in [9,16]). Type I SGs form upon exposure to various types of environmental stress (ER stress, oxidative and thermal stress), which is linked to global silencing of the canonical cap-dependent translation (initiation) and thus, they sequester large fraction of transcripts that are translationally silent [14]. In turn, transcripts mounting the stress response (such as those with ATF4-dependent expression [17]) are preferentially excluded from SGs to maintain stress response and cell survival [13]. Recent study, that employs single-molecule cell imaging, presents evidence about an active translation of ATF4-dependent in SG and a dynamic exchange with the cytoplasmic pool of translating mRNAs [18], thus, explaining the prevalent view of preferential recruitment and overrepresentation of non-translating mRNAs with the fact that mRNA accumulation in SGs correlates with the runoff time of translating ribosomes from mRNAs of different length [19,20]. The approach does not have the resolution to disentangle whether translation takes place throughout the whole SGs or at their surface, within the flexible outer SG layer, yet it supports the notion about highly dynamic SG structures actively exchanging with the cytosol.

In healthy cells, SGs are transient and disassemble following stress relief. Two major pathways of SG removal have been proposed: autophagy-dependent and autophagy-independent [21–23]. The former is used for clearance of SGs under long-lasting chronic stress, whereas the latter is consistent with recycling of SGs in response to short-lasting or acute stress exposure whereby mRNAs are protected and will re-enter translation after stress removal [21,22,24]. The disassembly mechanism may also vary dependent on the stress type. G3BP1, a core SG protein required to maintain the assembly of SGs, is ubiquitinated in SGs assembled under heat stress, but not in those formed under oxidative stress [25]. In turn, while ubiquitination is not required for heat shock-induced SG condensation, it is essential for their disassembly [22,25]. In yeast, SGs, that form under nutrient deprivation, disassemble in a metabolite-dependent manner by controlling the dynamic assembly and disassembly of the pyruvate kinase Cdc19, the core SG seeding component that assembles into amyloid aggregates and promotes SG formation [26]. While we are beginning to understand the SG disassembly and the variety of tightly controlled clearance, it remains unclear whether mRNAs deposited in SGs completely recover for translation following stress relief.

In this study, we identified the mRNAs recovering for translation using RNA-seq. Nearly 90% of the mRNAs sequestered in the SGs following acute stress exposure recovered for translation. In a previous study, we discovered that SGs constitute of two different types of mRNAs, unmodified or pervasively m^6^A-modified [14]. Monitoring the fate of these two groups, we observed that the methylated mRNAs recovered nearly fully for translation (96%), whereas from the non-modified a substantial fraction was lost (16%). Thus, the m^6^A modification may display some advantage to mRNAs, most likely owing to the m^6^A-driven structural stabilization in the near vicinity of the m^6^A modification.

## Results

### m^6^A-modified mRNAs disassociate first from SGs during stress relief

**Figure 1.**
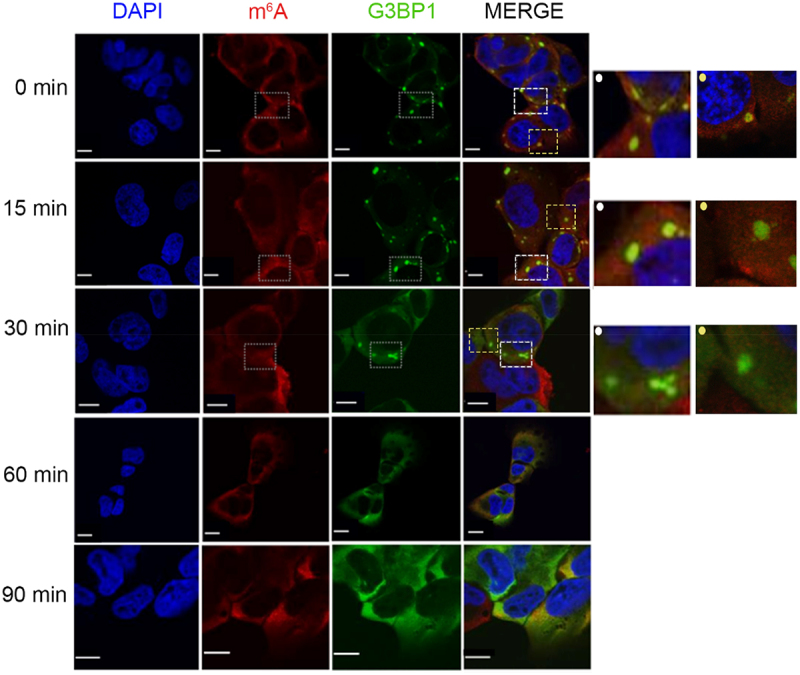
m^6^A-signal dissipates first followed by clearance of the SG cores at later time point. Time course of SG disassembly in U2OS-G3BP1-GFP cells monitored by confocal microscopy. Cells were pre-exposed to 500 µM AS for 30 min and allowed to recover in permissive growth medium and. Zero min denotes the time point of medium exchange and withdrawal of AS. SGs were visualized by G3BP1–GFP (green), m^6^A-modified mRNAs with m^6^A antibodies (red), nuclei were counterstained with DAPI (blue). Insets on the left, zoomed in area depicted on the merged image designated with dashed-line squares and a dot (left corner) in the corresponding colour. Scale bar, 10 µm.

In an earlier study, in which we addressed the SG assembly, we observed that mature SGs are formed within 30 min following exposure to 500 µM arsenie (AS) in U2OS and HEK cell lines [14]. In response to acute stress, the majority of the actively translated mRNAs are sequestered in the SGs; whereby non-modified mRNAs localized mostly in the SG core and methylated mRNAs decorate the surface of the SGs [14]. Using U2OS cells with endogenously GFP-tagged SG core protein G3BP1 [27], we recapitulated these observations: the m^6^A signal depicting the m^6^A-modified mRNAs was somewhat enriched at the surface of the SGs (Figure S1A and [14]). The different distribution of m^6^A-modified and non-modified mRNAs in the SGs [14] raised the question as to whether mRNAs will gradually leave SGs, with m^6^A-modified and surface-localized mRNAs being first. To monitor the dissociation pattern of SG mRNAs, we performed a time-course immunostaining following stress relief. U2OS-G3BP1-GFP cells were first exposed to acute oxidative stress (500 µM AS) for 30 min and then transferred to permissive growth medium. Already at 15 min following stress relief, the m^6^A signal in SGs significantly decreased, and at 30 mins it completely dissipated from the SGs (Figure 1). The SG core was still detectable as a dense hyperfluorescent loci by the scaffolding G3BP1 protein (Figure 1). At 90 min, the SGs completely vanished (Figure 1). Similarly, in another cell line (i.e. HEK293 cells stably expressing another SG marker, TIA1-GFP [28], thereafter named HEK-TIA1), the m^6^A signal of the SGs also dissipated first (Figure S1B). Together, these results suggest a gradual SG disassembly, with m^6^A-modified mRNAs and/or mRNAs localized at the SGs surface leaving first.

### SG-protected mRNAs recover nearly completely for translation

In our earlier publication, using m^6^A-seq and RNA-seq we identified that in HEK-TIA1 cells a very large fraction of the mRNAs in SG are methylated [14]. Using these sequencing data sets, we extracted the identities of all mRNAs detected in SGs (5,219 in total) and separated them into two groups: (1) 2,461 m^6^A-modified mRNAs containing at least one m^6^A modification within the DRACH motif (i.e. the conserved motif for m^6^A installation, with D = A/G/U, R = A/G and H = U/A/C [29]), and (2) 2,758 unmodified transcripts. Methylation patterns are highly conserved among eukaryotes [29,30] and the biological replicates displayed very high reproducibility (R^2^ > 0.9, Person coefficient [14]), yet the m^6^A position within the same transcript may vary between DRACH motifs in a close proximity, thus, by grouping the transcripts we used the transcript IDs and not the m^6^A position.

**Figure 2.**
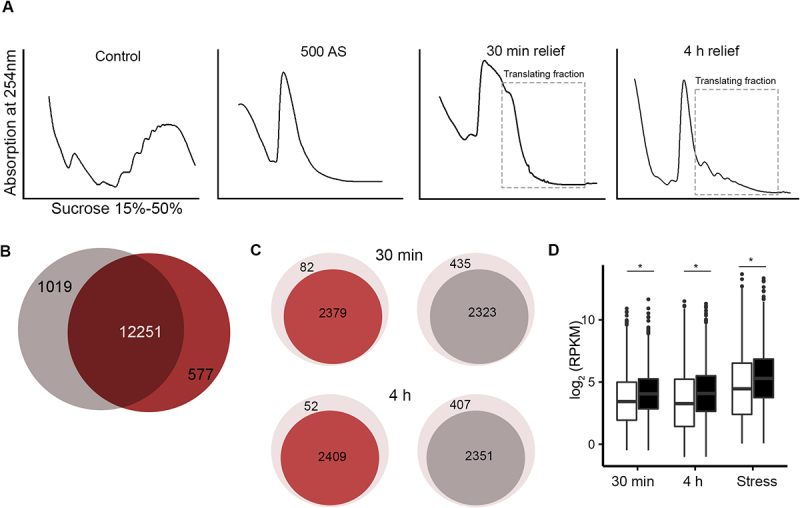
Nearly all mRNAs deposited in SGs are translated upon stress relief. (A) Translation profiles using sucrose gradients of HEK-TIA1 cells following stress recovery. Cells were pre-exposed for 30 min to stress (500 µM AS) and samples were collected at 30 min and 4 h of stress relief (i.e. after medium exchange and AS removal). Control denotes cells grown under permissive conditions and not being exposed to stress. Translating mRNA fractions, which were collected for RNA-seq, are designated. (B) Total number of unique mRNAs identified as translating (i.e. in the polysomes) at 30 min (grey) and 4 h (red) following stress relief. (C) Translating mRNAs at the two time points following stress relief (darker colours) whose identities overlap with m^6^A-modified mRNAs in SGs (red) and non-methylated mRNAs in SGs (grey) (D) Boxplot of the abundance (RPKM) of translating mRNAs. mRNAs are separated by their modification status in the SGs, i.e. m^6^A-modified (black) and non-methylated (white). Stress denotes expression of mRNAs isolated from cells exposed 500 µM AS for 30 min, and grouped based on their methylation status in SGs. p = 4.82x10^−22^, p = 1.17x10^−27^ and 2.07 × 10^−25^ Mann–Whitney test between methylated and non-methylated mRNAs at 30 min, 4 h and total mRNA, respectively. Note that for cells exposed to stress total mRNAs was analysed, hence, the higher expression levels than in cells after stress relief in which only the polysome-bound mRNAs were considered.

We next sought to determine whether these m^6^A modified and unmodified mRNAs pools that were sequestered in SGs would recover for translation upon stress relief. We first compared translation profiles using sucrose gradients (Figure 2(a)). Acute stress (500 µM AS) leads to complete inhibition of translation, i.e. complete loss of the heavy polysomal fraction (Figure 2(a)). At the time point, at which the m^6^A signal completely dissipated from the SGs (at 30 min relief), some heavy polysomal fractions appeared, suggesting that cells resumed some translation activities, albeit very poor. Active translation was detectable after the complete SG dissociation (at 4 h, Figure 2(a)), although to much lower extent than the translation in unstressed cells, implying that much longer times are needed for complete recovery from stress.

Next, we collected both the monosomal and polysomal fractions at 30 min and 4 h in HEK-TIA1 cells, extracted the total RNA by hot-acid phenol and subjected the mRNAs to RNA-seq. Usually, heavy polysomes are considered as genuinely translating ribosomes, but increasing evidence suggests that monosomes are also translating [31,32], hence, we pooled monosomes and polysomes, which henceforth are named translating mRNAs. A total of 13,207 and 12,828 unique transcripts were detected in the samples collected at 30 min and 4 h, respectively; the identified mRNAs between both time points were largely overlapping (Figure 2(b)). Native SGs are fragile and do not co-migrate with polysomes; only crosslinked and stabilized SGs can be separated with sucrose gradients [14], implying that the identified translating mRNAs are genuinely translating species. Strikingly, the majority of all transcripts identified previously to be protected in SGs were found in the translating fraction following stress relief (Figure 2(c)), corroborating the notion of the protective effect of SGs on mRNAs during acute stress [33,34]. We compared the identities of the translating mRNAs at both time points following stress relief with those found in SGs as methylated (group 1) and non-methylated (group 2). Both methylated and non-methylated transcripts were present in the translating pool at 30 min or 4 h after stress relief (Figure S2A, B). However, a higher fraction of the mRNAs found to be methylated in SGs (96% at 30 min and 97% at 4 h) recovered for translation, compared to the non-methylated transcripts (84% at both time points) (Figure 2(c)). Methylated mRNAs were slightly longer (i.e. with an average length of 3.2 kb) than the non-methylated transcripts with an average length of 2.5 kb (Figure S2C-F); the length difference was majorly reflected by differences in the length of their 3'UTRs. In addition, the methylated mRNAs were significantly more abundant in the translating fraction than the non-methylated ones (Figure 2(d)). The same difference in the expression pattern, with modified mRNAs being more stable than the unmodified, was detected in the cells exposed to stress (Figure 2(d)).

The m^6^A modification is reversibly installed by a ‘writer’ complex (methyltransferase like 3 [METTL3], methyltransferase like 14 [METTL14], and Wilms’ tumour 1-associating protein [WTAP]) and reversed by demethylases termed ‘erasers’ (fat mass and obesity-associated protein [FTO] and AlkB homologue 5, ALKBH5) [29,30,35], all of which localized in the nucleus. Oxidative stress does not cause any redistribution of the writers and erasers [14], thus, we can exclude alterations of the methylation pattern of SG mRNAs directly in the cytosol. Since mRNAs are m^6^A modified exclusively in the nucleus in a co-transcriptional manner, we reasoned that a *de novo* methylation of newly synthesized transcripts could be the reason for the higher fraction of methylated transcripts among the translating mRNAs. To address this, we inhibited the *de novo* transcription by treating the cells with a sublethal dose of actinomycin (ActD) [36] during the recovery time, isolated translating mRNAs from the polysomes and subjected them to RNA-seq. At 30 min and 4 h upon ActD treatment, we identified the same transcripts as in HEK-TIA1 cells not treated with ActD (Figure 2(b)), i.e. 12,725 and 12,593 transcripts, respectively (Figure S2G). Similar to the untreated cells with ActD (Figure 2(c)), a higher fraction of mRNAs was found to be methylated in SGs (98% at both time points) recovered for translation, compared to the non-methylated transcripts (89% at both time points) (Figure S2H). When comparing the distribution of both mRNA groups found in SGs following ActD treatment, the distribution of both methylated and unmethylated remained unchanged compared to untreated cells (Figure S2I compare to Figure 2(c)), all together arguing against the contribution of de novo methylation to the higher fraction of methylated transcripts among translating mRNAs.

Together, these results suggest that m^6^A modification may display some advantage to the mRNAs in their recovery for translation. The effect could be direct and affect the mRNA stability as shown earlier [37,38], and/or indirect as the higher transcript abundance or larger transcript length may simply correlate with the higher probability of being methylated.

### mRNA is more structured in the near vicinity of m^6^A modification

**Figure 3.**
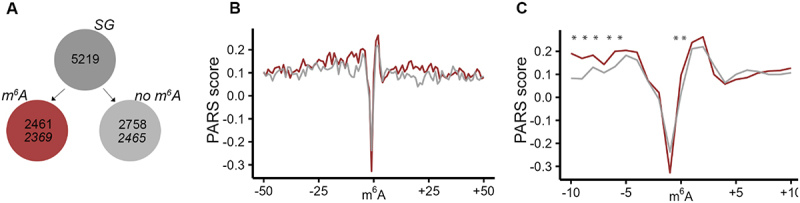
mRNAs exhibit higher structural propensity in the m^6^A vicinity. (A) From the SG mRNA clients nearly all methylated (red) and non-methylated (grey) mRNAs were detected in the PARS data set (italicized numbers). (B) Aggregated PARS score plotted centred at the m^6^A including 50 nt up- and downstream of the transcripts. (C) Zoom in into 10-nt window up- and downstream of the m^6^A. Red, m^6^A-modified mRNAs; grey, non-modified mRNAs. *, p < 0.05, Mann-Whitney test.

m^6^A modification regulates mRNA stability [38] and is a main driver of mRNA turnover [39]. Our observation that higher fraction of m^6^A-modified mRNAs recovered for translation, raised the question as to whether this could be due to the m^6^A-dependent structure stabilization of the transcript. To assess the m^6^A effect on the intrinsic propensity of mRNAs to form secondary structure, we considered a published parallel analysis of RNA structure (PARS) data set [40]. PARS is based on fragmentation of isolated, protein- or ribosome-free mRNA with structure-specific enzymes, e.g. a double strand-specific RNase V1 or a single strand-specific S1 nuclease. Coupled to deep sequencing, PARS reports on the intrinsic propensity of each nucleotide within a transcript to partition in secondary structure [41]. Across tissues and cell types of humans, methylation patterns are constitutively maintained and regulatory secondary structures conserved [39,42–44], thus, we considered the published PARS analysis of the isolated mRNA from a model human cell line, HepG2 [40] as representative for the human mRNA structure. We computed the PARS score for each nucleotide (i.e. log_2_ ratio between the normalized reads from the RNase V1-treated and the S1 nuclease-treated samples) and compared them for the transcripts of the two groups, i.e. with m^6^A modification and non-modified mRNAs (Figure 3(a)). A positive PARS score indicates higher propensity of a nucleotide to be involved in a secondary structure, and vice versa, lower PARS score indicates no involvement of the nucleotide in a secondary structure. In the group of methylated mRNAs, we plotted the PARS score within the vicinity of methylated DRACH motifs. From the non-modified mRNA set, we selected mRNA segments with unmethylated DRACH motifs, thus, comparing sets with identical nucleotide signature in the closest m^6^A vicinity. The m^6^A methylation alone markedly decreased the PARS score at the modified A nucleotide (Figure 3(b,c)), suggesting decrease of the structure propensity at the m^6^A nucleotide, which corroborates earlier observations [45]. This effect was not limited to nonmethylated mRNAs found in the SGs, but was uniform for all other mRNAs with a DRACH motif in the transcriptome (Figure S3). However, it should be noted that the effect of the methylation scored statistically not significant, most likely because the A nucleotide in the non-methylated DRACH motifs exhibited also a very high intrinsic propensity to be unstructured, i.e. very low PARS score (Figure 3(b,c)). Intriguingly, we observed a significant increase in the secondary structure propensity (i.e. increase of the PARS score) of the nucleotides in the immediate vicinity of m^6^A (Figure 3(c) and S3). Together, this analysis suggests that while m^6^A modification decreases structure at the modified adenine, but enhances structuring at nucleotides in the immediate vicinity of the methylated adenine, that in turn may increase the mRNA stability, at least locally.

## Discussion

Here, we address an important aspect of the dynamics of SG disassembly, namely the recovery of mRNAs from SGs. We observed a gradual recovery of the SG-sequestered mRNAs with methylated mRNAs being among the first to leave the SGs. Combining fractionation of translating ribosomes with RNA-seq-based identification of mRNAs in translating pools, we detected that the majority of the SG mRNAs recover for translation, with modest but significantly higher recovery of m^6^A-modified mRNA compared to non-methylated ones, i.e. 95% vs 84%, respectively. The recovery of translating mRNAs, both modified and unmodified, does not change upon inhibition of *de novo* transcription, supporting the notion that we detect the redistribution of SG mRNAs into translating fraction.

Deep sequencing-based structural PARS analysis shows that at the N^6^ modification punctually enhances the tendency of the modified adenine to be more single-stranded, corroborating thus earlier observations [45–47]. By contrast, m^6^A renders the structural propensity of the nucleotides in its nearby vicinity and we found them with enhanced ability to participate in secondary structures. m^6^A can destabilize RNA duplex by 1.4 kcal/mol [48] and alter locally mRNA structure, thereby exposing RNA binding motifs and facilitating binding [45,49]. In some other contexts, however, m^6^A can contribute to stabilization of secondary structures. For example, m^6^A-U pair facilitates RNA secondary structure via canonical Watson-Crick geometry and by stabilizing adjacent base pair by adding a favourable hydrophobic interaction [50]. Thus, m^6^A modifications may act as conformational switch or structural remodeller and through stabilizing or destabilizing local secondary mRNA structures may modulate interactions with RBPs. In the mRNA group, which we detected as modified in the SGs, the m^6^A modification is the highest in the vicinity of the stop codon and 3'UTRs [14]. Higher structuring at 3'UTRs, likely mediated by m^6^A modification in this region, correlates with poor targeting by miRNA-mediated mRNA degradation and higher stability of the modified mRNAs [51,52]. Recent more precise gene-level quantification of m^6^A positions suggest a strong contribution of m^6^A modification to mRNA stability and mRNA half-life and link it directly to the steady-state mRNA levels [39]. mRNAs with longer half-life times are more pervasively m^6^A modified [39]. Thus, based on the observation for a much higher or nearly complete recovery of m^6^A-modified mRNAs for translation compared to non-modified mRNAs, it is conceivable to propose that m^6^A provides an advantage to mRNAs in their recovery for translation by likely increasing the mRNA’s stability through m^6^A-driven structuring in the close vicinity of the modification site.

## Materials and methods

### Cell lines, growth conditions and immunostaining

U2OS cells stably expressing GFP-tagged G3BP1, a SG marker [27], were used for immunofluorescence staining. HEK293 cells expressing N-terminally FLAG tagged SG marker TIA1 (or HEK-TIA1) [28] were used to perform all sequencing experiments. Both cell lines were grown in DMEM medium at 37°C, 5% CO_2_. To induce oxidative stress, 500 µM sodium arsenite (AS) was added to the medium for 30 min. To recover from stress, medium was exchanged with fresh medium without AS. To inhibit de novo transcription, cells were treated with ActD (0.5 µg/mL; stock solution 1 mg/ml in DMSO) [36], which was kept in the medium throughout the stress recovery time. Note than control cells were treated the same way, i.e. subjected to same medium changes and incubation times without the stress agent.

For immunostaining, U2OS cells were grown directly on coverslips. For imaging, cells were washed twice with PBS, fixed for 15 min with 4% paraformaldehyde at room temperature, and permeabilized using 0.5% saponin. Subsequently, cells were blocked with 1% BSA in PBS for 1 h at RT and incubated with the primary m^6^A antibody (1:200 dilution; Synaptic Systems) for 1 h at RT. After washing with PBS, cells were incubated with AlexaFluor 568-labelled secondary antibody (1:200 dilution) for 1 h at RT and imaged on Leica-TCS-SP5 confocal microscope, on one Z-plane. Images were processed by ImageJ with FIJI plugin.

### Polysome profiling, RNA isolation and RNA-seq library preparation

10–15 million cells were harvested at 850xg and resuspended in 500 µl cell lysis buffer (10 mM Tris-HCl (pH 7.4), 5 mM MgCl_2_, 100 mM KCl, 1% Triton X-100, 2 mM DTT and 100 μg/ml cycloheximide) and subjected immediately to lyses. Cells were shear opened with 26-gauge needle by passing the lysate through it 8-times. It should be noted that cells were not preincubated with cycloheximide to stall elongating ribosomes, known to cause some distortion in the ribosome profiling [53]; cycloheximide is only added post lysis to stabilize ribosome-mRNA complexes in the processing steps. 400 µL of the cell lysate was loaded onto 5-ml sucrose gradient (60% to 15% sucrose) dissolved in polysome buffer (50 mM HEPES-KOH, pH 7.4, 5 mM MgCl_2_, 100 mM KCl, 2 mM cycloheximide, 2 mM DTT) and separated by ultracentrifugation at 148,900xg (Ti55 rotor, Beckman) for 1.5 hours at 4°C.

Polysome fractions were collected and total RNA was extracted from each fraction by adding 0.1 volume of 10% SDS and one volume of acidic phenol-chloroform (5:1, pH 4.5), incubated at 65°C for 5 min, and centrifuged at 21,000xg at 4°C for 5 min to separate different phases. Equal volume of acid phenol-chloroform was added to the aqueous phase, separated by centrifugation and supplemented with an equal volume of chloroform:isoamyl alcohol (24:1). Upon separation, the aqueous phase was supplemented with 0.1 vol 3 M NaOAc (pH 5.5) and an equal volume of isopropanol, precipitated for 3 h at −20°C, RNA was pelleted at 21,000xg at 4°C, and the dried pellets were resuspended in DEPC-H_2_O. The purified RNA was fragmented in alkaline fragmentation buffer (0.5 vol 0.5 M EDTA, 15 vol 100 mM Na_2_CO_3_, 110 vol 100 mM NaHCO_3_) and subjected to cDNA library preparation as described [54].

### Sequencing analysis

Sequenced reads were trimmed by *fastx-toolkit* (0.0.13.2; quality threshold: 20) and first depleted from the adapter sequences using *cutadapt* (1.8.3; minimal overlap: 1 nt). Only reads uniquely mapping to the human reference genome (GRCh38.p13) using STAR [55] (2.5.4b) allowing one mismatch (–outFilterMismatchNmax 1 – outFilterMultimapNmax 1) were considered. From the parsed genome annotation files, we considered the longest isoform for genes containing more than one transcript isoform. Mapped reads were normalized as reads per kilobase per million mapped reads (RPKM).

CLIP-seq and m^6^A-seq were downloaded from [14] and used to determine the SG clients and methylated mRNAs, respectively. The analyses were performed as described earlier [14]. Each sequencing set consist of two libraries, i.e. RNAs immunoprecipitated with m^6^A-antibody (IP sample) and total RNA before subjected to immunoprecipitation (input sample). Briefly, for each transcript in the IP sample the peak over median (POM) was determined using a sliding window of 50 nt (with a 25-nt overlap). The POM is calculated by dividing the mean coverage within the sliding window by the median coverage across the entire transcript. Windows with a POM score higher than three and minimal mean coverage of ten were retained. The input sample was also subjected to the same POM score analysis. Shared regions between the IP and input samples were discarded as false-positives. For the remaining POM within each window, we calculated the ratio of the scores from the IP sample to the input samples resulting into a peak over input (POI) score. DRACH motifs were predicted using HOMER algorithm [56]. POIs detected only within DARCH motifs were selected. Transcripts containing minimum one methylated DRACH motif, i.e. minimum one POI within a DRACH motif, were considered as methylated and counted to the group of methylated transcripts. All other transcripts, with no POI within a DRACH motif, were considered as non-methylated.

The PARS data sets were downloaded and analysed as described [38]. Briefly, each data set contains two libraries produced from the same total RNA, i.e. one treated with RNase V1 which cleaves double-stranded RNAs and the other with nuclease S1 which recognizes single-stranded regions. Trimmed reads were uniquely aligned to the human reference transcriptome (ENSEMBLE GRCh38.p13) using Bowtie (1.2.2) allowing one mismatch. Reads were normalized to the size of the corresponding libraries as reads per million (RPM). The PARS score is computed as described [57], which is defined as the log_2_ ratio of the normalized reads (RPM) from the RNase V1-treated and the S1 nuclease-treated samples.

## Supplementary Material

Supplemental Material

Supplemental Material

## Data Availability

RNA-seq of mRNAs engaged in the polysomes, that were generated in this study, are deposited in the Gene Expression Omnibus (GEO) under the accession number GSE189099. PAR-CLIP and m^6^A-Seq data sets were downloaded from the BioSample data base under accession number SRP121376. PARS data set was downloaded from GEO repository under the accession number GSE70485.
