# Supplementary material for: mRNAs sequestered in stress granules recover nearly completely for translation
Source: RNA Biol. 2022 Jul 7;19(1):877–84. doi: 10.1080/15476286.2022.2094137 (PMC9272840; doi:10.1080/15476286.2022.2094137)
Supplement: Supplemental Material [file KRNB_A_2094137_SM9625.pdf]

## Supplementary Information

# mRNAs sequestered in stress granules recover nearly completely for translation

Sarada Das[a], Leonardo Santos[a], Antonio Virgilio Failla[b], Zoya Ignatova[a]*

[a]Institute of Biochemistry and Molecular Biology, University of Hamburg, Hamburg. Germany.

[b]Microscopy Imaging Facility, University Medical Center Hamburg-Eppendorf, Hamburg, Germany

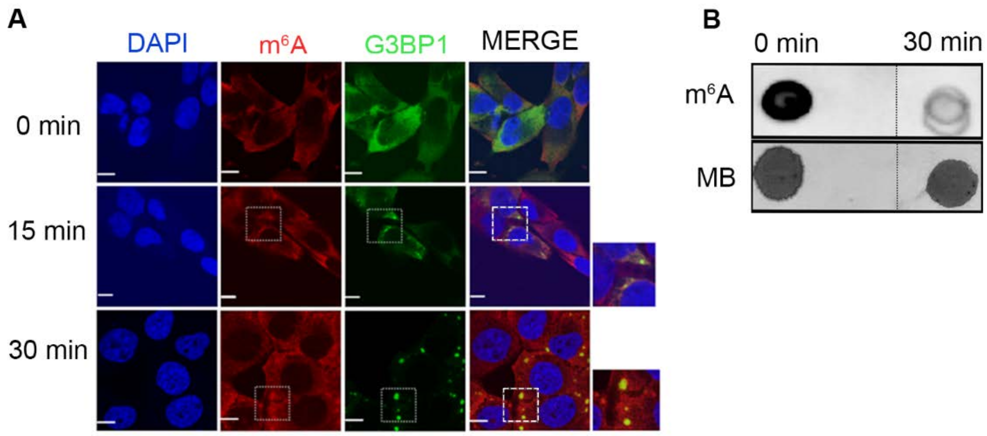


**Figure S1. SGs readily form in U2OS-G3BP1 cells in response to oxidative stress.** (A) Time course of SG formation of U2OS-G3BP1-GFP cells exposed to 500 µM AS monitored by confocal microscopy. Already at 15 min a thin rim of m$^6$A signal around the G3BP1-positive SG foci was detectable, which increased at 30 min. SGs were visualized through the scaffolding G3BP1–GFP protein (green), m$^6$A-modified mRNAs with m$^6$A antibody (red); nuclei were counterstained with DAPI (blue). Insets on the left, zoomed in area depicted on the merged image (dashed-line white squares). Scale bar, 10 µm. (B) The m$^6$A signal decreased in SGs isolated from HEK293-TIA1-GFP cells 30 min post-exposure to 500 µM AS, suggesting a decrease of the m$^6$A-modified mRNAs in SGs. Zero time point denotes the medium exchange and withdrawal of AS. MB, methylene blue staining of the total mRNA. Thin vertical lanes denote excised samples unrelated to this experiment.

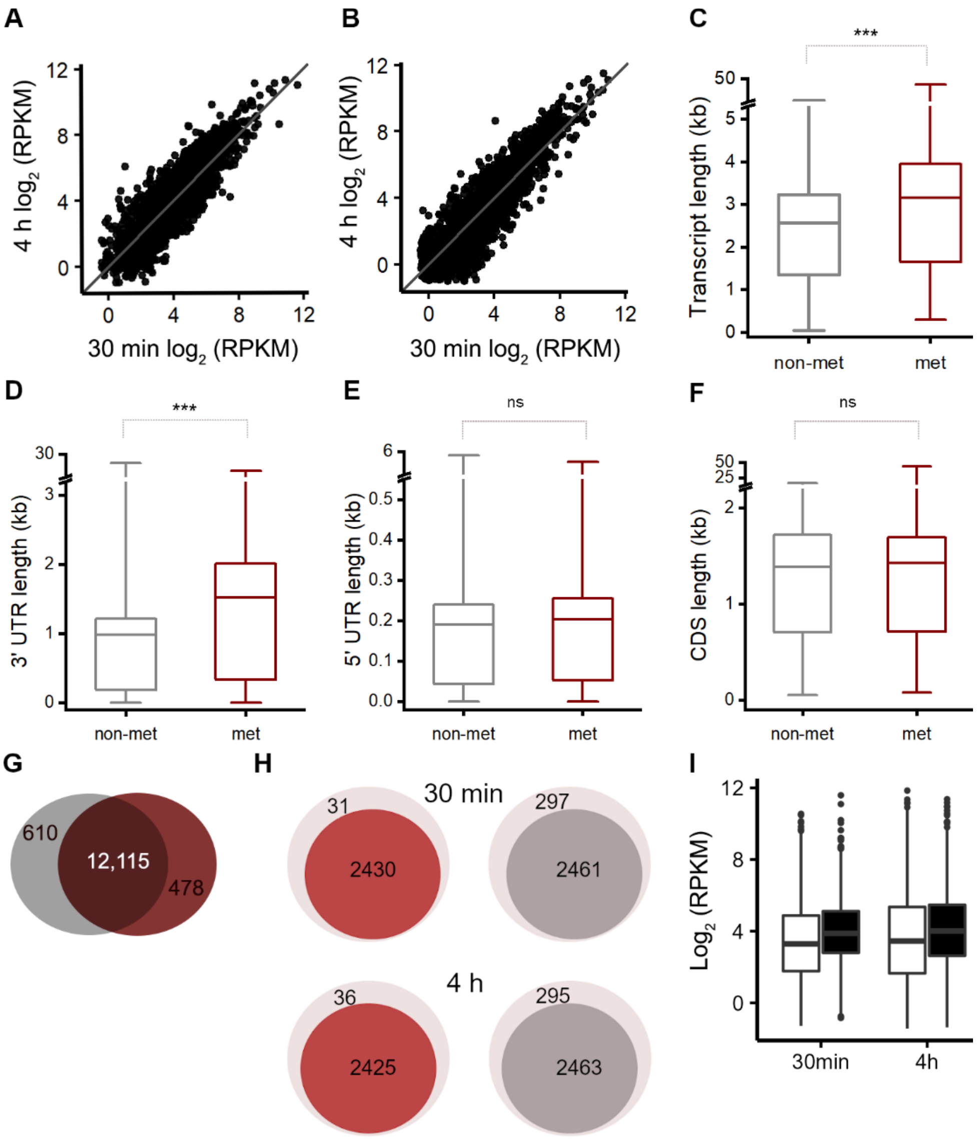


**Figure S2. mRNAs abundance analysis of translated mRNAs during stress recovery.** (A, B) Scatter plot comparing the abundance of translated mRNAs in HEK-TIA1 cells at 30 min vs 4 h following stress relief. The abundance (RPKM) is determined by RNA-seq. The identified mRNAs were separated based on their methylation status in SG, i.e. m$^6$A-modified (A) and non-methylated (B) transcripts. (C-F) Box plots comparing sequence features of non-methylated (non-met) and methylated (met) mRNAs (A, B), e.g. whole transcript length (C), 3'UTRs (D), 5'UTRs (E) and CDC (F). **, $p > 0.01$; ***, $p > 0.001$; ns, non-significant (Student's *t* test). (G) Venn diagram of translating mRNAs identified at 30 min (gray) and 4h (red) of HEK-TIA1 treated with ActD during stress relief. The abundance (RPKM) is determined by RNA-seq. (H) Translating mRNAs at two time points following ActD treatment and stress relief (darker colors) whose identities overlap with m$^6$A-modified mRNAs in SGs (red) and non-methylated mRNAs in SGs (grey) (I) Boxplot of the abundance (RPKM) of translated mRNAs in HEK-TIA1 treated with ActD. mRNAs are separated based on their methylation status in SG, i.e. m$^6$A-modified (black) and non-methylated (white) mRNA. $p = 3.07\times10^{-23}$ and $p = 2.23\times10^{-16}$; Mann–Whitney test between methylated and non-methylated mRNAs at 30 min and 4 h, respectively.

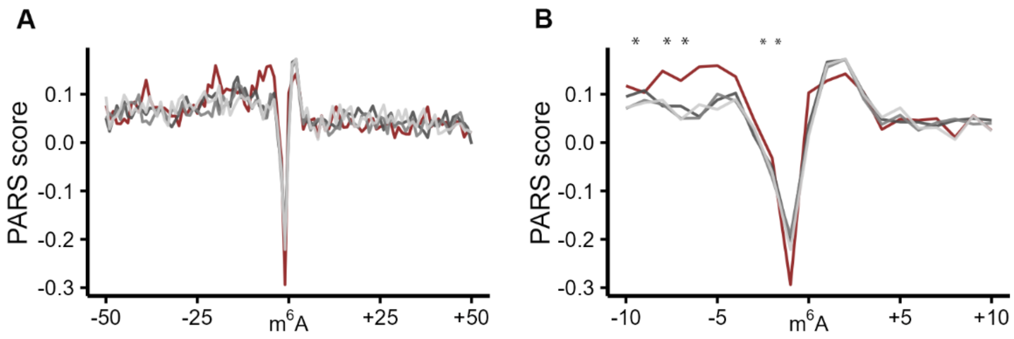


**Figure S3. Sequences in immediate proximity of the m$^6$A exhibit higher structure propensity.** (A) Aggregated PARS score plotted as centered at the m$^6$A including 50 nt up- and downstream of the m$^6$A site. Since in the whole cellular transcriptome the m$^6$A-modified mRNA set (red) was much smaller than the non-modified mRNAs, the latter was randomly split into three groups of a similar size (three shades of gray) to the m$^6$A-modified ones. (B) Zoom into 10-nt window up- and downstream of the m$^6$A site. Color code as in panel A.*, $p < 0.05$, Mann-Whitney test.
